# Understanding the Corrective Effect of the Urban Growth Boundary Policy on Land Finance Dependence of Local Governments in China

**DOI:** 10.3390/ijerph19084785

**Published:** 2022-04-14

**Authors:** Wentao Niu, Ting Nie, Xiao Chen, Tianxi Wang, Jingyi Shi, Zhenzhen Xu, Hexiong Zhang

**Affiliations:** 1 School of Management, Zhengzhou University, Zhengzhou 450001, China; yovennyting@gmail.com (T.N.); chenx@gs.zzu.edu.cn (X.C.); shi__jingyi@163.com (J.S.); 2 Business School, University of Edinburgh, 29 Buccleuch Place, Edinburgh EH8 9JS, UK; s1819714@ed.ac.uk; 3 School of Architecture and Built Environment, Deakin University, Geelong 3219, Australia; xuzhenz@deakin.edu.au; 4 College of Public Administration, Central China Normal University, Wuhan 430079, China; 15333868696@163.com

**Keywords:** the urban growth boundary policy, land finance dependence, the corrective effect

## Abstract

The preference for land urbanization of local governments promotes urban sprawl, which leads to the dilemma of land finance dependence (LFD) of local governments and the negative constraints on the ecosystem of urban areas in China. However, how the urban growth boundary (UGB) policy corrects local governments’ reliance on land finance has not been discussed in depth. In July 2014, the UGB policy began to be piloted in fourteen cities in China, providing a setting to further reveal the effectiveness of the UGB policy. By constructing an evolutionary game simulation model to clarify the behavioral strategies that local governments tend to adopt in the context of the UGB policy implementation, this study proves that the effective implementation of the UGB policy, by controlling the urban land capacity, can help solve local governments’ LFD dilemma in China. The UGB policy consists of a set of technical means and policy tools that controls urban sprawl. It breaks the “unlimited land capacity” situation faced by local governments in China by limiting the urban land capacity within a given period of time, and has become a new solution to the dilemma of LFD. The implementation of the UGB policy highlighted the shortage of urban land, which has led to the increasing cost of land finance for local governments and constraints on local governments’ LFD behavior. The shortage has also forced local governments to adjust and optimize their fiscal revenue structure. The UGB policy induced ongoing evolution in the benefit distribution among relevant entities in land finance, motivated local governments and other entities to adjust their primary strategies, and made it possible to address the dilemma of LFD in China.

## 1. Introduction

As a response to the reflection of urban sprawl, the concept of urban growth boundary (UGB) originated in the United States [1]. The specific concept of the UGB was first proposed by Salem, Oregon in the United States, that is “the UGB is the boundary between urban land and rural land”, and other scholars defined the UGB from different perspectives [2,3]. In the 1970s and 1990s, the UGB policy was placed at the core of the policy system in the “smart growth” period of urbanization in the USA. Although urban economists have been long skeptical about the urban suppression policy [4], the UGB policy has been widely adopted in the United States and most western countries today. Early research on the UGB policy mainly focused on the motivation and its correction of “urban sprawl”. The motivation of the UGB policy can be summarized as “protecting urban space, preventing urban sprawl and promoting urban intensive development” [2]. As the policymaker, the government considers whether to adopt the UGB policy based on its financial constraints and the policy’s potential demonstration effect. In general, cities with adjacent cities that have implemented the UGB policy and cities with fewer fiscal constraints (i.e., wealthier cities) are more likely to adopt the UGB policy [5]. In addition, urban growth also has negative externalities. To mitigate such externalities, urban growth management policies such as the UGB policy are essential [6].

For a long time, “whether the UGB policy can correct the trend of urban sprawl” has been a research hotspot. The State of Oregon, which began to implement a strict UGB policy in 1973, has become an important object of relevant research. Most studies on Oregon show that the implementation of the UGB policy has effectively changed urban ecology and played an important role in restricting urban sprawl [7]. This result is mainly realized by restricting the urbanization of agricultural, forest, and other non-agricultural land outside the range of the UGB. For research on this topic, a more critical issue is how to measure the degree of urban sprawl. The existing research is mainly based on population density [8,9], housing construction scale [10], and other urban attributes before and after the implementation of the policy.

In recent years, relevant studies have turned to evaluating the effect of the UGB policy, including the impacts of the UGB policy on land value, real estate market, and the spatial heterogeneity in the policy effect [11]. Some researchers find that the UGB policy has no significant impact on the real estate market and urbanization rate [12,13], while some researchers hold an opposite view and believe that the UGB policy has a significant impact on land value, housing price, urban built-up area and land development potentials [14,15]. Nevertheless, the extent to which the UGB policy can modify “urbanization inertia” is relatively limited in the short term [16]. The spatial heterogeneity in the effect of the UGB policy has also been widely discussed.

In areas where the UGB policy has been implemented, there are spatial differences in the response to the policy inside and outside the urban growth boundary, as evidenced by relevant studies by Dempsey et al. (2013) [16] and Gennaio (2008) [17]. Dempsey (2013) answered the relationship between “the UGB policy and its restriction on urbanization expansion” through the analysis of the UGB policy in Oregon, United States based on multiple estimation, and believed that the UGB restrained urbanization development in most cities of Oregon. Gennaio et al. (2008) [17] evaluated the effectiveness of the urban boundary policy in Switzerland by analyzing the indicators of land development, building expansion, and urban building density in Swiss cities from 1970 to 2000. They think that the implementation of the UGB policy restrains the development of the urban built-up area inside the UGB and intensifies its building density, while the building density outside the UGB boundary decreases. Currently, relations among the UGB policy, urban transportation [18,19], and agricultural land [20] are also research hotspots on this topic.

After the implementation of “Methods for Urban Planning Formulation” by the Chinese Ministry of Construction in 2006, a large number of studies by Chinese scholars on the UGB policy began to emerge, and they experienced a transformation from introducing the implementation background and connotation of the UGB policy in the US during the early stage [21] to emphasizing the sinicization of the UGB theory in the later stage. The existing research focuses on the method of delimitation of urban boundary, the rigid and elastic principle of urban boundary, and the sinicization of the UGB theory [22,23]. Considering the UGB is essentially an inner variable of urban economy, the level of a specifically defined boundary is inevitably determined by the economic operation of a city. As a result, the determination and dynamic adjustment of the UGB should consider not only the direct cost, but also the economic benefits caused by the UGB and the comprehensive carrying capacity of the city [21].

A reasonable urban growth boundary can effectively prevent the disorderly spread of cities, situate the urban growth space in the most feasible area, and provide proper guidance for cities’ future development [24]. Some scholars take “controlling urban land expansion and protecting cultivated land” as the criterion of applicability of the UGB, and choose “quantity control and boundary reference” as the demarcation method of the UGB [25]. Other scholars emphasize the coordinated analysis of “spatial evaluation of urban comprehensive carrying capacity, simulation results of urban spatial expansion and important ecological spatial distribution”, and the division of UGB by combining natural and historical boundaries [26,27].

Once the UGB is delimited, it cannot be changed in the short term, implying its rigidity. However, it can be adjusted within a certain range through scrutiny and approval procedures when necessary [21,28], and this indicates UGB’s elasticity. The term of elasticity emphasizes the process, while rigidity focuses on the ultimate goal [29]. As the UGB has certain technical, policy, and dynamic characteristics, it is reasonable to classify the rigid boundary and elastic boundary under the influence of the dynamic mechanism and the constraint mechanism generated by economy, society, and environment [24]. The UGB management mode cannot be simply copied to China, but must be customized from four aspects: proper positioning, delineation method, integrated management, and flexible control [30]. There are two reasons for the sinicization of the UGB theory: one is the fundamental difference in policy environment between China and the US, such as the difference in urbanization stage and land ownership; the other one is the dynamic difference of urban sprawl and urban space expansion in China and the US [31,32]. These differences imply different actions in the UGB policy. The relations among the UGB policy, urban overall carrying capacity [33], ecology [34,35], and urban spatial organization [36,37] also interest Chinese scholars.

In general, scholars are concentrated on the implementation effect and mechanism of the UGB policy. They agree with the inhibitory effect of the UGB policy on urban sprawl and emphasize revising this policy in the implementation process. It is a basic consensus that the UGB should be adjusted moderately. However, the corrective mechanism of the UGB policy on urbanization process has not been thoroughly studied. China has now entered the stage of accelerated urbanization process in which land urbanization is significantly faster than the population urbanization. Considering that local governments are trapped in the dilemma of “city scale infatuation” and LFD, as well as the inefficient scale expansion of urban space, China’s urbanization process is currently facing many negative constraints [38].

China’s urban spatial management system is mainly based on policy-specific and use-specific spatial designations. The policy of space designation in China is subject to the control requirements of regional economic development and resource coordination, and the space can be divided into “optimized development zones, key development zones, restricted development zones and prohibited development zones” [39]. Meanwhile, spatial uses can be classified into four main categories: residential area, industrial area, roads and squares, and green spaces. Specific uses and proportions of each category depend on the spatial classification requirements of various levels of management. The UGB policy is currently used as one of China’s planning tools for optimizing the extent of urban planning areas, for deepening and integrating policy space delineation and for defining development space in urban areas [40]. Before entering the land exchange market, the supply of urban construction land and residential land in China is mainly under the responsibility of local governments. Specifically, local governments, through unified land acquisition, demolition, resettlement, compensation, as well as the construction of basic supporting facilities within a certain area, bring the land to a level where it can be used directly and then enter the market circulation as a special commodity.

In 2014, the Chinese government began to make strategic arrangements for the UGB policy system, and successively issued several policy texts on the “delimitation of urban growth boundary”. The policy description on the demarcation of urban growth boundary in “The National New Urbanization Plan (2014–2020)” was issued on 16 March 2014. The “pilot work on demarcation of urban development boundary” carried out by the Ministry of Land and Resources and the Ministry of Housing and Urban-Rural Development in fourteen large cities in July 2014 also includes the strategic deployment of the comprehensive demarcation of permanent basic farmland in the “Central Document No. 1” (the first policy statement released by central authorities each year) in 2015. To a certain extent, all these arrangements constitute the key content of China’s UGB policy system. Since 2014, the UGB policy has been implemented in fourteen cities in China (these cities include Beijing, Shanghai, Shenyang, Nanjing, Hangzhou, Xiamen, Zhengzhou, Wuhan, Guangzhou, Shenzhen, Chengdu, Guiyang, Xi’an, and Qingdao). The experiment has been ongoing for more than seven years, which provides a realistic basis for the systematic discussion on how the UGB policy can correct local governments’ preference in city size and dependence on land finance. This study also provides new evidence for the effectiveness of the UGB policy.

## 2. Realistic Background

With the rapid expansion of urban space in China, the scale of land finance revenue (LFR) and land finance dependence (LFD) in thirty-five large and medium-sized cities in China has been continuously growing since 1999. LFD is measured by the proportion of LFR in local governments’ fiscal revenue structure, and the calculation method can be obtained from our earlier research [41,42]. From 1999 to 2017, among thirty-five large and medium-sized cities, Kunming had the highest annual growth rate of LFR, reaching 52.29%. In 1999, Kunming’s LFR was only CNY 23 million. In 2017, this index value increased to CNY 44.233 billion. In 2011, Kunming’s LFR even reached CNY 72.264 billion. Eleven cities had an annual growth rate of LFR that fluctuated between 40% and 50%. These cities are: Wuhan (49.42%), Zhengzhou (47.35%), Xining (46.32%), Ningbo (43.96%), Nanchang (43.77%), Jinan (43.42%), Hefei (42.26%), Shijiazhuang (41.87%), Chengdu (40.73%), Hohhot (40.38%), and Qingdao (40.15%).

Thirteen cities had an annual growth rate of LFR that fluctuated between 30 and 40%, including: Shenyang (39.63%), Taiyuan (38.28%), Fuzhou (37.26%), Hangzhou (36.52%), Chongqing (34.94%), Changsha (34.86%), Haikou (34.21%), Urumqi (33.40%), Guiyang (32.93%), Nanjing (32.00%), Harbin (31.87%), Lanzhou (30.07%), and Yinchuan (31.02%). The average annual growth rate of LFR fluctuated between 20 and 30% in seven cities, including Tianjin (27.45%), Xi’an (27.54%), Changchun (27.27%), Xiamen (27.10%), Nanning (22.78%), Shanghai (21.75%), and Beijing (21.16%). Only three cities saw an annual growth rate of LFR below 20%, including Shenzhen (19.11%), Guangzhou (16.17%), and Dalian (14.27%). It is noteworthy that although the average annual growth rate of Dalian’s LFR was only 14.27% from 1999 to 2017, the index value reached CNY 104.22 billion in 2011, and then dropped to only CNY 14.138 billion in 2017, resulting in a seemingly low average annual growth rate in the whole observation period. Overall, in the observation period, the LFR of thirty-five large and medium-sized cities all showed a rapid growth process.

The fiscal and tax decentralization system established by the tax-sharing reform since 1994 is the main cause of the LFD dilemma of local governments in China. The urban-rural dual land system is the key institutional basis for the land finance dilemma. At the same time, the asymmetrical cost-benefit balance of land expropriation, the centralized distribution mechanism of land revenue to local governments, and the lack of fairness of land revenue distribution among local governments of different generations further aggravate the dilemma of LFD of local governments. The evolution of the LFD provides an intuitive description of the land fiscal dilemma of local governments. Before the real construction of the unified land factor trading market, the only way for China’s rural collective land to participate in the market trading is that “the transformation process of rural land from collective ownership to nationalization”.

In the expropriation of rural collective land, local governments and their agents often have significant bargaining power compared with farmers. Farmers, under most circumstances, can only be price takers in land expropriation. The expropriated individuals who reject the land expropriation price are often regarded as dissenters by local society as “nail households”. Therefore, although the land expropriation price policy has been continuously reformed and optimized at the national level, the actual compensation received by farmers is often relatively low, and the low land acquisition price constitutes the main cost of state-owned urban construction land transfer for local governments.

Once the land is expropriated, it enters the nationalization process and finally enters the urban construction land trading market waiting for sale. After the conversion of land ownership, local governments sell rural collective land as urban construction land for substantial gains, regardless of whether their future land ownership becomes real estate project construction or not, and local governments can obtain considerable incremental revenue compared with the price of land expropriation. In the case of the above serious cost-benefit asymmetry, “land expropriation—land transfer—land fiscal revenue—urban spatial expansion—land urbanization path” will inevitably become the urbanization mode of local governments [39]. Therefore, behind urban sprawl, vested interest groups continue to maximize their benefit in all urban space elements, and land-lost farmers and other recipients of urban space rights allocation are constantly facing the lack of fairness.

From 1999 to 2017, the LFD rate of thirty-five large and medium-sized cities showed a declining trend. The maximum LFD rate occurred in Haikou in 2005, reaching 90.00%, and the index value decreased to 35.30% in 2017, the average LFD rate of Haikou was 29.75%. The maximum value of the average LFD rate that occurred in Hangzhou was 46.62%. In most years, the LFD rate in Hangzhou remained above 50% (in 2003, 66.71%; in 2004, 51.45%; in 2007, 56.98%; in 2009, 67.26%; in 2010, 60.43%; in 2013, 60.23%; and in 2017, 61.12%). Hefei (42.90%) and Nanchang (40.42%) also had an average LFD rate of more than 40%.

The average LFD rate of most cities fluctuated between 30 and 40%. There are eighteen such cities in total, including: Fuzhou (39.96%), Nanjing (39.09%), Ningbo (38.30%), Nanning (38.15%), Chengdu (38.04%), Jinan (37.37%), Xiamen (36.75%), Chongqing (35.16%), Wuhan (34.41%), Changchun (34.40%), Shenyang (33.64%), Dalian (33.61%), Changsha (33.52%), Shijiazhuang (33.47%), Qingdao (32.26%), Yinchuan (31.36%), Xi’an (31.30%), and Kunming (30.99%). There are eleven cities with average land finance dependence rates between (20 and 30%), including: Haikou (29.75%), Tianjin (29.56%), Harbin (28.02%), Zhengzhou (27.71%), Taiyuan (27.68%), Guangzhou (26.87%), Beijing (25.92%), Guiyang (25.64%), Xining (25.41%), Hohhot (25.23%), and Lanzhou (23.95%).

Only three cities, Shanghai (19.31%), Urumqi (17.13%), and Shenzhen (15.44%), had an average LFD rate below 20%. In 1999, the dependence rate of Shanghai’s LFR was 9.05%, which peaked at 30.79% in 2004 and then steadily declined to 18.26% in 2017. Beijing has a similar pattern. In 1999, the dependence rate of Beijing’s LFR was 23.52%, and peaked at 45.89% in 2004, then steadily declined to 33.36% in 2017. It is noteworthy that although the LFD rate in Shanghai and other cities was relatively low, the absolute scale of LFR in Shanghai is extremely large. In 2013, the scale of land finance revenue in Shanghai exceeded CNY 100 billion, and then reached CNY 160,881 billion in 2015. In general, the LFR scale and LFD rate of thirty-five large and medium-sized cities in China have been continuously growing, which basically outlines the realistic picture of LFR preference, LFD dilemma, and land urbanization mode preference of local governments in China.

## 3. Theoretical Framework and Research Hypothesis

The tenure of Chinese government officials is short, and an important way that officials are evaluated is based on GDP, the two features that have intensified economic competition among local governments. At the same time, the tax-sharing reform changed the means of competition among local governments, making them rely on differentiated land concession strategies for different land uses and the expropriation of rural land for a “scissors gap” of profits [42]. Land transfer fees and land mortgage financing of local governments constitute an important complement of fiscal revenue, promote urban infrastructure construction and public service level, and strengthens regional economic growth [43], which is consistent with local governments’ eagerness to “sell land to attract investment”. The empirical study shows that land finance is an important driving force of economic growth, but the relation is not monotonic, as excessive LFD impedes economic growth.

At present, the theoretical framework and empirical research on the relationship between land finance and economic growth have deepened. Scholars have become increasingly interested in the relationship between land finance and urban industrial structure, urban functional layout, and urban production efficiency. In differentiated land transfer strategies, local governments offer appealing conditions such as low land price or even zero land price to enterprises to attract investment. This induces practical problems such as too much idle land and inefficient use of urban industrial land. Therefore, studies on land finance and urban production efficiency are increasingly abundant, aiming to clarify the logical relationship between land finance and urban efficiency, improve land use efficiency, and regional productivity, and promote the sustainable growth of regional economy. Due to different resource endowments in different regions, the impact of land finance on urban output efficiency or total factor productivity is heterogeneous.

Some scholars point out that the expansion of urban construction area caused by land finance has a negligible impact on urban total factor productivity [44]. However, land finance influences urban total factor productivity due to local governments’ differentiated strategies in land sales. The low-cost transfer of industrial land strategy can improve the total factor productivity of the city, while the high-cost transfer of land for residential and commercial strategy can produce a negative effect [45]. The low-price transfer of industrial land has become an important strategy for local governments to obtain an edge in land competition, which causes waste in land resources and a mismatch of urban resources, and further leads to urban sprawl.

As the land monopoly supplier, local governments place a large amount of land into the real estate development market for LFR, which booms the real estate industry. Local governments’ differentiated land transfer means of selling commercial and residential land at a high price and industrial land at a low price has promoted industrial development to a certain extent [46,47]. Therefore, the mode of the “horizontal subsidy” gap between commercial or residential land prices and industrial land prices is constantly strengthened and has become a common method used by local governments. Local governments’ preference for land finance is growing because local governments are not only the policymakers of land expropriation and the direct executors of the land expropriation process, but also the monopolists of the primary land market.

Local governments reduce the cost of land for investment enterprises through low or even zero land prices to attract more funds to support the development of the regional economy. On the one hand, the development of industrial enterprises can provide a lot of tax revenue to the local governments; and on the other hand, the production of industrial enterprises can provide a lot of productive and living materials and sufficient jobs for the cities. Many scholars have found that industrial development and progress have promoted urban construction and increased urbanization levels [48,49]. Although the low-price supply of industrial land comes with a certain loss of land value, the increase in tax revenue and the high-price supply of commercial and residential land can compensate the loss. As the rational “economic man”, local governments rely on the differentiated land sale strategy to enhance local financial power, which accumulates wealth for improving urban public infrastructure construction to a certain extent.

However, studies show that, facing the imbalance between fiscal power and authority brought by fiscal decentralization, local governments make up fiscal revenue through land sale fees, but the push for urban construction has not been accompanied by an effective improvement in the level of urban public services [50]. The high prices of commercial and residential land sold by local governments do not affect the enthusiasm of real estate developers. The developers transfer high land prices to buyers and trigger an increase in house prices [51]. The rapid rise of housing price to income ratio weakens the affordability of urban residents, and intensifies the housing plight of low-income groups, migrant workers, and other groups with housing difficulties [52]. The high profits of real estate attract more speculative capital into the real estate industry, resulting in inefficient allocation of social resources and low urban productivity [53].

More land development stimulates a further expansion of urban space. In terms of land type, land under control of local governments usually includes land for public facilities, industrial land, residential land, green land, and special land. The impact of land finance on urban space expansion in China can be classified into the following aspects:

On the one hand, the industrial land fee is relatively low, but in the long run, industrial land can provide constant tax revenue for local governments [54]. Local governments over-consume industrial land and reduce the land use price endlessly to attract enterprises and capital in competition with other local governments. Local governments expect the capital can develop industrial parks and construct economic development zones to further boom the economy and attract more developers and investors. However, the above strategies lead to excessive depletion of urban land. Urban development is a phased process, and is also a long-term virtuous cycle. A large number of industrial parks with an unsaturated and low utilization rate reduces the utilization rate of urban land and even leaves it abandoned.

On the other hand, commercial land, (e.g., residential land), can produce one-off revenue for local governments. In order to keep the fiscal balance, local governments try to restore the loss from the low prices of industrial land by transferring larger commercial land and selling commodity residential land through auction, bidding, and trading, etc. Therefore, this pattern naturally induces unnatural market fluctuations in urban real estate prices [55]. In addition, residential areas that are far away from downtowns become scattered and partially clustered living communities with low density. The consequent living style of residents then stimulates the continuous expansion of urban space.

The rapid increase in land finance provides important financial guarantee for local urban infrastructure construction, improves urban public services, and basic social insurance, and creates opportunities for future development. However, it is also an important factor leading to the expansion of urban space [56].

Facing strong market competition, local governments must face a series of stress, such as land income, performance appraisal, and financial pressure. Thus, it is necessary to control the valuable assets and apply a reasonable transfer strategy. Local governments compete with peers to attract land developers for more investment and provide subsidies when necessary. Market investment is profit-seeking, and facing the preferences of local governments, investors tend to invest more in the preferential projects. In this sense, the government needs to keep coming up with more preferential strategies. Such competition, to a large extent, becomes the so-called “land competition” [57].

According to the general law of economics, when land supply is fixed, with an increase in land demand, the cost of land expropriation by local governments will also increase, and the rent-seeking space in local governments’ “land finance” will be greatly compressed, forcing them to reduce their LFD and look for new sources. Unlike central cities and suburban urbanization in western countries, in China the “GDP worship” among Chinese local officials and the financial pressure brought by the tax-sharing reform are the main drivers behind cities’ blind expansion [32]. The demarcation of urban development boundary does not intervene market function, but helps to balance market supply and demand. Therefore, in the context of urban boundary control and intensive use of urban land, local governments generally face the transformation of urban land use mode from “incremental land” development to “stock land” development and reuse. Therefore, this study proposes Hypothesis 1 as follows:

**Hypothesis** **1.** *The preference for land urbanization of local governments promotes the blind expansion of urban space, which leads to the dilemma of LFD of local governments. The implementation of the UGB policy forces local governments to rationally change their land use patterns, which is conducive to the final resolution to the dilemma of LFD*.

## 4. Identification of LFD Interest Groups

### 4.1. The Central Government

The central government represents national interests, and the land ownership, land management, and land expropriation systems in China are all formulated and supervised by it. In recent years, with the disordered expansion of urban boundaries, a large amount of cultivated land has been expropriated. Although the system of “expropriation and compensation” has been implemented in some areas, the phenomenon of “expropriating more and compensating less”, “expropriating the good and compensating the bad”, “expropriating the land and compensating the mountain” and so on, are increasingly common. China has a large population but a shortage in arable land resources. Urbanization at the expense of cultivated land has raised concerns on national food security.

The disordered expansion of the urban fringe destroys the ecological landscape such as grassland and forest in suburbs, and destroys the ecological environment around the city. In addition, the land system formulated by the central government stipulates that only through expropriation can rural land enter the market to transfer its land-use rights. However, the current land expropriation system stipulates that the compensation for agricultural land is only six to ten times the average annual output value of the past 3 years. Land is the base of farmers’ lives, and agricultural output is relatively low and is greatly affected by natural conditions. Low land compensation fees deepen the poverty among land-lost farmers.

In order to ensure national food security and protect the legitimate rights and interests of farmers, the central government formulated a series of policies to protect farmland and classify some permanent basic farmland, unswervingly adhere to the bottom line of ecological protection, and limit the boundary for urban growth. On the other hand, at the end of 1998, China abolished the welfare housing allocation policy. The repeal comprehensively promoted the market-oriented construction of housing and created a large housing demand in the market. At the same time, the country’s tax-sharing reform continues, and the land finance strategy can also help to meet the housing market demand for land to some extent. The resulting outcome is the rapid development of the real estate market. In this sense, the various systems and policies of the central government may have two goals: promoting economic growth, and maintaining social equity.

There are two ways through which the central government can regulate local governments. The first way is centralization, the central government can strengthen its supervision on local governments and scrutinize any possible illegal behavior of local governments. This can discipline the behavior of local governments, reduce their LFD, protect people’s legitimate rights and interests, but is likely to be costly. Another way is decentralization, that is, the central government relaxes the supervision and control on local governments, leaving local governments more incentives to develop their economy. Nevertheless, this can also harm the fairness of social development [58].

### 4.2. Local Governments

Local governments are the frontline executors of the policy from the central government, and they are also under the supervision of the central government. Local governments should adhere to the central government’s bottom line of 1.8 billion mu of farmland, while also pursuing local economic development and people’s interests. Therefore, in the policy implementation, the preferences and decisions of local governments are the key factors in the effect of policy implementation [59]. Although China’s land system is formulated by the central government, the granular land expropriation and land transfer are completed by local governments, which are the monopolistic expropriator and supplier of land. Considering China’s decentralized fiscal system and official performance assessment system, local governments have a strong incentive to participate in land expropriation in order to gain an advantage in inter-regional competition and governors’ administrative promotion [60].

Therefore, local governments tend to balance two goals. One is to develop local economy. Local governments can provide financial support and subsidies to gain an advantage over other regions in the competition. However, too much LFD might be detected by the central government. Another goal is to promote personal interests, that is, the purpose of local governments’ participation in urbanization construction and promotion of economic development is for governors’ personal promotion. If governors fail to achieve promotion, they will hope to obtain economic compensation through rent-seeking with their political power.

### 4.3. Real Estate Developers

Real estate developers are on the demand side of land-use rights, and they become suppliers of commercial and residential products after real estate development and construction. In order to maximize their benefit, local governments supply industrial land at low prices to attract investment, and in turn supply residential and commercial land at high prices to make up for the loss in “land attracting investment”. Real estate developers act in accordance with homo-economicus, and they tend to turn the high land price into the housing price. Consequently, house buyers are the ultimate bearers of the cost. Real estate developers can acquire land-use rights through bidding, auction, or listing on the land market, and the use rights are sold to the developers offering the highest price.

In order to obtain the land-use rights of a certain period and complete the strategic deployment of the enterprise, real estate developers may also seek rent to increase the probability of obtaining the land-use rights or bid even higher than their reserve price. Facing local governments’ monopolistic supply on land and the soaring land price, real estate developers may conspire to collectively raise the house price and infringe the interests of consumers, but they face the risk of administrative punishment.

### 4.4. Farmers Whose Land Has Been Expropriated

Urbanization is the transformation of rural land into urban land. Land expropriation is the basis and key of land finance. In land expropriation, the land compensation and resettlement subsidies provided by local governments to farmers are far below market standards. Land-lost farmers have little power to bargain and can only passively accept the offer, which does not allow them to share the benefit of land appreciation. Although land expropriation can be an opportunity for land-lost farmers to become urban residents and monetary compensation or materialistic compensation seem to increase farmer household wealth, the value of the compensation is overall low. The economic surplus loss seems to be transferred to real estate developers, but it eventually turns into a high house price for house buyers.

In this process, the wealth of land-expropriated farmers is far from enough for them to settle down in urban areas, resulting in the impoverishment of land-lost farmers. Farmers are aware of the difficulties stemming from land expropriation, so they try to strive for more compensation from local governments. If the negotiation does not go well, they may take a confrontational attitude toward local governments, which may even induce social conflicts.

## 5. Multidimensional Game Analysis of Land Finance Dependence Dilemma

This study focuses on the corrective effect of urban boundary control policy on local governments’ dependence on land finance, and analyzes the decisions and behaviors of each stakeholder from the perspective of game evolution theory. Game theory is widely used to explain real-life situations with competition among different stakeholders. In the game evolution analysis, the “replicative dynamics” of organisms are used to model their evolutionary and dynamic adjustment processes. This process can be characterized by a replicated dynamics equation, which is a dynamic differential equation that describes the frequency with which a group adopts a certain strategy. In this section, a set of dynamic equations of the evolutionary game among the relevant subjects is constructed based on the average expected returns of the subjects in the game, and then the Jacobi matrix of these equations is derived. The stable state is confirmed according to the relevant results of the Jacobi matrix, and if the dynamic equation system reaches the stable state in the iteration, the strategy can be called an evolutionary stable strategy.

### 5.1. Game Analysis between Central Government and Local Governments

#### 5.1.1. Model Construction

The central government emphasizes intensive and economical use of land and proper protection of cultivated land. Relevant policies formulated by the central government need to be carried out by local governments, and measures should be taken to check the implementation status of local governments. Given the shortcomings of China’s official promotion system and land supervision system, local governments have a strong incentive to seek land finance by violating central government’s regulations. Since there is no binding agreement between the central government and local governments, local governments can reap huge land wealth without any punishment if their violation is undetected.

Therefore, there is a non-cooperative game between the central government and local governments, and whether the land system and policies of the central government can be implemented depends on how strongly the central government supervises and regulates local governments for possible violation. The central government requires local governments to implement the land policy, but local governments are reluctant to implement the policy due to their own benefit. Local governments may still rely on land finance to raise profit and maximize their own interests. At this point, the central government will launch a review mechanism to increase the punishment on local governments for violation.

In general, the central government’s strategic choice set is supervision or not supervision; local governments’ strategy choice set is no violation or violation. The implementation of the UGB policy is one of the situations in which the central government’s strategy is “supervision”.

#### 5.1.2. Model Assumptions

**Assumption** **1.** *In the absence of supervision by the central government, when the land-related policy of the central government is implemented by local governments, the central government’s income is*$R_{0}$*; in the absence of supervision, the income from illegal operations of local governments is*$R_{1}$*. As the central government performs the role of maintaining social fairness and maximizing social welfare,*$R_{0}$*is inevitably greater than*$R_{1}$.

**Assumption** **2.** *When the central government loosens its supervision, local governments conscientiously and strictly implement the land policy of the central government, and the revenue is*$R_{2}$*and local governments pursue their own interests maximization, and the revenue is*$R_{1}$*. Without the constraint of the central government, local governments revenue*$R_{1}$*is surely greater than*$R_{2}$*, but the illegal operation of local governments bring serious negative externalities to the society, such as urban disease, rent-seeking, corruption, and other problems*.

**Assumption** **3.** *The cost paid by the central government to mitigate negative externalities caused by local governments’ violation is*$C_{0}$*, the penalty imposed by the central government on local governments’ violation is*P*, and the portion of illegal gains confiscated by the central government is*S*. In order to avoid the illegal operation of local governments, the central government’s supervision cost of local governments is*$C_{1}$.

#### 5.1.3. Interest Matrix and Evolutionary Game Model

x represents the probability that local governments will choose not to violate the regulations, 1−x represents the probability that local governments violate the rules, y represents the probability that the central government will not monitor, and 1−y represents the probability that the central government will monitor. And the game profit matrix of the central government and local governments is shown in Table 1.

(1) Evolutionary game dynamic equation of local governments

According to the model assumptions, when local governments *D* adopt the strategy of “no violation” and “violation”, the expected returns and average returns are $U_{11}$, $U_{12}$, and $U_{1}$, respectively, which are calculated as follows:

(1) $$\begin{array}{l} U_{11}=yR_{2}+(1-y)R_{2}=R_{2}\\ U_{12}=yR_{1}+(1-y)(R_{1}-P-S)=(y-1)(S+P)+R_{1}\\ U_{1}=xU_{11}+(1-x)U_{12}=(1-x)(y-1)(S+P)+x(R_{2}-R_{1})+R_{1} \end{array}$$

The replication dynamic equation is a dynamic differential equation that can be used to describe the frequency of which a particular strategy is employed by a population. The evolutionary game replication dynamic equation of probability x that the local governments choose the “no violation” strategy is:

(2) $$F_{D}(x)=dx/dt=x(U_{11}-U_{1})=x(1-x)(R_{2}-R_{1}-yS-yP+S+P)$$

(2) Evolutionary game dynamic equation of the central government

The expected return and average return when the central government Z adopts the strategy of “no monitoring” and “monitoring” are $U_{21}$, $U_{22}$, and $U_{2}$, respectively, which are calculated as follows:

(3) $$\begin{array}{l} U_{21}=xR_{0}+(1-x)(R_{0}-C_{0})=(x-1)C_{0}+R_{0}\\ U_{22}=x(R_{0}-C_{1})+(1-x)(R_{0}-C_{0}-C_{1}+P+S)=(x-1)C_{0}+(x+1)(P+S)+R_{0}-C_{1}\\ U_{2}=yU_{21}+(1-y)U_{22}=(y+1)(xP+xS+P+S-C_{1})+(x-1)C_{0}+R_{0} \end{array}$$

The evolutionary game replication dynamic equation of the probability y that the central government chooses the “no monitoring” strategy is:

(4) $$F_{Z}(y)=dy/dt=y(U_{21}-U_{2})=y(1-y)(C_{1}-xP-xS-P-S)\;$$

Copy the dynamic equation system composed of Equations (2) and (4), as shown in Equation (5):

(5) $$\begin{array}{l} F_{D}(x)=dx/dt=x(U_{11}-U_{1})=x(1-x)(R_{2}-R_{1}-yS-yP+S+P)\\ F_{Z}(y)=dy/dt=y(U_{21}-U_{2})=y(1-y)(C_{1}-xP-xS-P-S) \end{array}$$

(3) Evolutionary equilibrium analysis of cooperative subject behavior game

Let the equation value of the replicated dynamic Equation (5) be zero, and five equilibrium points are obtained, namely A (0, 1), B (0, 0), C (1, 0), D (1, 1), and E $\left(C_{1}/(P+S)-1,(R_{2}-R_{1})/(S+P)+1\right)$.

The Jacobian matrix obtained from the replicated dynamic equations of Formula (5) is:

(6) $$\left[\begin{array}{cc} F1 & F2\\ F3 & F4 \end{array}\right]$$

According to the assumptions, any initial point and its evolved point are meaningful in the two-dimensional space V={(x,y)|0≤x≤1,0≤y≤1}, so $C_{1}<2P+2S,R_{2}<R_{1}$. Set the determinant of the matrix as det(J) and the trace of the matrix as  tr( J), the stability analysis of the five equilibrium points is shown in Table 2.

Because the value of the trace of the equilibrium point E is 0, the equilibrium point B and the equilibrium point D do not satisfy the situation of det( J)>0,tr( J)<0, so E ($C_{1}/\left(P+S\right)-1,(R_{2}-R_{1})/\left(S+P\right)+1$), B (0, 0), D (1, 1) does not satisfy the evolutionary stable strategy conditions. The conditions for the remaining two equilibrium points to become local stable points are shown in Table 3.

#### 5.1.4. Stability Analysis of Equilibrium Points

(1) A (0, 1)

Point A (0, 1) means the local governments violate the rules and the central government does not monitor it. When $P+S<C_{1}$, it is the only equilibrium point. This situation usually occurs in the early stage of the implementation of the land policy, when the central government’s penalties for local governments’ illegal operations and the confiscation of illegal benefit is lower than the cost for the central government’s monitoring on local governments. Due to high monitoring cost, local governments are less pressured by the central government, and the benefit of violation is relatively large. Thus, A (0, 1) denotes the situation in which the local governments violate the rules and the central government does not supervise.

Obviously, point A (0, 1) is not the expected outcome of the UGB policy implementation. This situation can be improved by increasing P, S and decreasing $C_{1}$. The implication of point A (0, 1) is that the central government not only needs to increase the penalties for local governments’ illegal operations, but also needs to increase the proportion of confiscation of illegal profits. Moreover, the central government should improve the supervision system, reduce the cost of supervision, establish a normalized supervision system, and increase the intensity and breadth of supervision on local governments.

(2) C (1, 0)

Point C (1, 0) means the local governments do not violate the regulations and the central government supervises. When $R_{1}-P-S<R_{2},C_{1}<2P+2S$, it is the only equilibrium point. This situation usually occurs during the implementation period of the land policy, when the central government’s supervision measures for local governments are enhanced, and the supervision system is feasible and effective. In this case, the relevant benefit of the local governments to implement the central government’s policy are higher than the benefit of violation, and the condition of $R_{1}-P-S<R_{2}$ can be achieved. In this case, local governments choose to implement the policy under the supervision of the central government, which is an ideal win-win situation for the central government and local governments.

#### 5.1.5. Evolution Simulation Analysis

In order to further reveal the evolutionary game relationship between the central government and local governments, and figure out the conditions for the two to reach an evolutionary equilibrium, MATLAB 2020b software (developed by MathWorks, Natick, MA, USA) was used to simulate and analyze the above situation.

**Figure 1:**
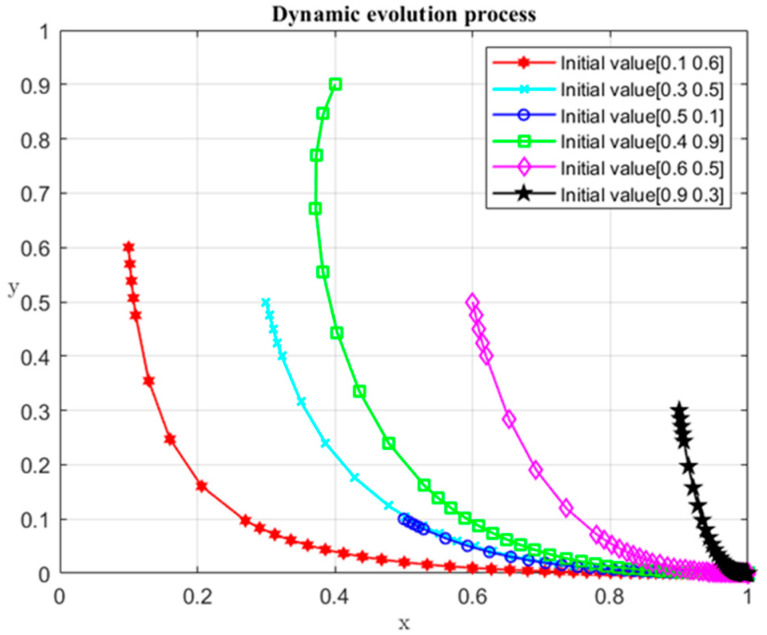
The dynamic evolution process of the strategy choice (the central government and local governments).

It can be seen from the above game process that when $R_{1}-P-S<R_{2}$ and $C_{1}<2P+2S$, the system evolves to the desired equilibrium point (1, 0). Detailed information of parameters is shown in Table 4, and the initial values (x, y) of the numerical simulation are set as (0.1, 0.6), (0.2, 0.8), (0.3, 0.5), (0.5, 0.5), (0.6, 0.5), (0.7, 0.2), (0.9, 0.3), and (0.7, 0.8). The dynamic evolution process of participant strategy selection over time is shown in Figure 1. It can be seen from Figure 1 that under the specified conditions, no matter what the initial values are, the system transforms to (1, 0). The (1, 0) represents the case where the local governments choose the non-violation strategy and the central government chooses the monitoring strategy.

(1) The impact of local governments’ illegal operation revenue $R_{1}$ on the evolution results

**Figure 2:**
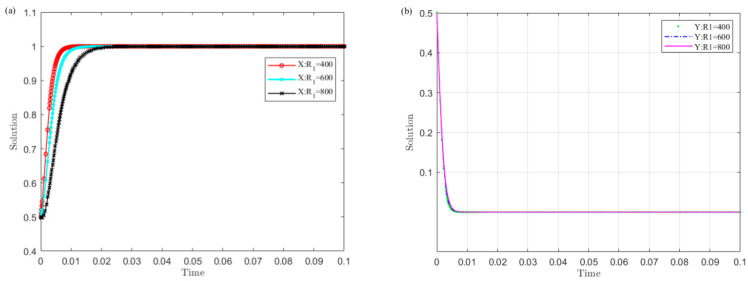
System strategy evolution results for $R_{1}=400,\;R_{1}=600,\;R_{1}=800$. (**a**) local governments; (**b**) the central government.

Set $R_{1}$ as 400, 600, 800, and simulate the strategy evolution of the central government and local governments. The results are shown in Figure 2.

Figure 2 shows that the smaller the $R_{1}$ value of local governments’ illegal operation revenue, the sooner local governments converge to the non-violation strategy and the central government chooses the monitoring strategy. Among them, local governments are more sensitive to the changes in $R_{1}$ than the central government. This shows that reducing the income of local governments’ illegal operations is conducive to the successful implementation of the UGB policy.

(2) The impact of local governments’ implementation of relevant policy benefit on the evolution results

**Figure 3:**
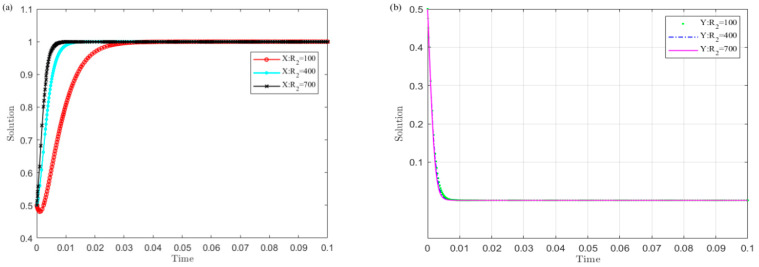
System strategy evolution results when $R_{2}=100,\;R_{2}=400,\;R_{2}=700$. (**a**) local governments; (**b**) the central government.

Set $R_{2}$ as 100, 400, and 700 and simulate the evolution of the central government’s and local governments’ strategies. The results are shown in Figure 3.

The trends in Figure 3 show that the larger the $R_{2}$ value of local governments’ implementation of the policy, the sooner local governments converge to the non-violation strategy and the central government chooses the monitoring strategy. Among them, the local governments are more sensitive to the changes in $R_{2}$ than the central government. This shows that increasing the benefit of local governments’ implementation of the policy is conducive to the successful implementation of the UGB policy.

(3) The influence of the central government’s supervision cost $C_{1}$ on the evolution results

**Figure 4:**
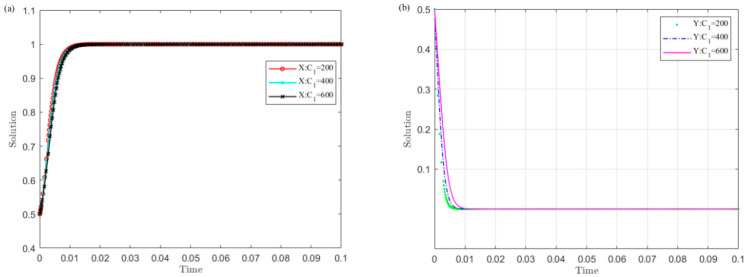
System strategy evolution results when $C_{1}=200,\;C_{1}=400,\;C_{1}=600$. (**a**) local governments; (**b**) the central government.

Set $C_{1}$ as 200, 400, and 600, and simulate the strategy evolution of the central government and local governments. The results are shown in Figure 4.

Figure 4 shows that the smaller the value $C_{1}$ of the central government’s supervision cost on local governments, the sooner local governments converge to the non-violation strategy and the central government chooses the supervision strategy. Among them, the central government is more sensitive to changes in $C_{1}$ than local governments. This indicates that reducing the monitoring cost is conducive to the successful implementation of the UGB policy.

(4) The impact of the central government’s punishment on local governments’ illegal operation P on the evolution results

**Figure 5:**
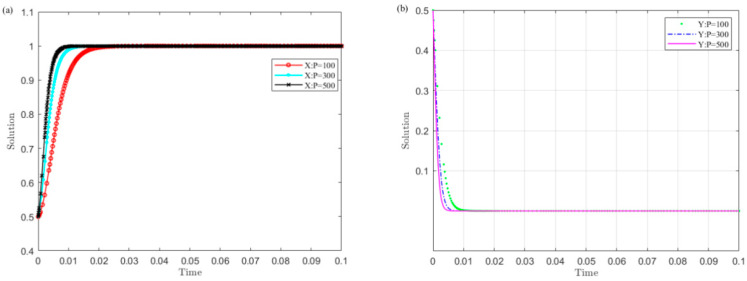
System strategy evolution results when P=100, P=300, P=500. (**a**) local governments; (**b**) the central government.

Set P as 100, 300, and 500, and the strategy evolution of the central government and local governments is simulated. The results are shown in Figure 5.

Figure 5 illustrates that the larger the central government’s penalty P value on local governments’ illegal operations, the sooner local governments converge to the non-violation strategy and the central government selects the monitoring strategy. Among them, the central government is more sensitive to changes in P than the central government. This implies that increasing the central government’s penalties on local governments’ illegal operations is conducive to the successful implementation of the UGB policy.

(5) The influence of the central government’s confiscation on local governments’ illegal revenue S on the evolution results

**Figure 6:**
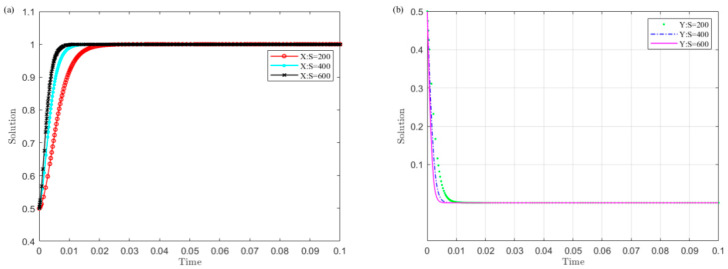
System strategy evolution results when S=200, S=400, S=600. (**a**) local governments; (**b**) the central government.

Set S as 200, 400, and 600, and the simulation of the strategy evolution of the central government and local governments is carried out. The results are displayed in Figure 6.

It can be found in Figure 6 that the greater the central government’s confiscation on local governments’ illegal revenue S, the sooner local governments converge to the non-violation strategy and the central government chooses the monitoring strategy. Among them, the local governments are more sensitive to change in S than central government. This indicates that increasing the central government’s confiscation on local governments’ illegal earnings S is conducive to the successful implementation of the UGB policy.

#### 5.1.6. Conclusions

Based on the payment matrix of the central government and local governments (Table 1), several findings can be concluded here. First, in the (supervision by the central government, violation by local governments) strategy, due to information asymmetry, when local governments actively implement the policy of the central government, the central government may still closely supervise local governments to ensure the effect of the policy. The cost of supervision by the central government is $C_{1}$, and the net revenue by the central government is $R_{0}-C_{1}$. The payoff for local governments is $R_{2}$. Second, in the (no supervision by the central government, no violation by local governments) strategy, the central government fully trusts local governments’ executive ability and working attitude. As a result, the central government relaxes its supervision, and the local governments still conscientiously perform their duties and actively implement the central government policy. At this point, the central government’s revenue is $R_{0}$ to maximize social welfare, and the local governments will also obtain a certain revenue of $R_{2}$. In reality, such an ideal equilibrium is rare.

Moreover, in the (supervision by the central government, violation by local governments) strategy, due to the reform of the national tax distribution system, local governments’ financial power is matched in authority, so the local governments rely on LFR to mitigate their financial shortage. Despite the central government’s increasing scrutiny on local governments and harsh penalties on their transgressions, local governments are still betting on the chance to cash in on their land and compete with other regions. In this case, the revenue of local governments is $R_{1}-P-S$, and the central government conducts urban governance, personnel punishment, and partial confiscation on illegal income from local governments’ violation. Therefore, the revenue of the central government is $R_{0}-C_{0}-C_{1}+P+S$. Finally, local governments are reluctant to strictly implement the central government’s policy due to the “opportunity cost” of the strategy. The central government’s lack of supervision on local governments further strengthens the dependence of local governments on land finance. In this case, the central government’s income is $R_{0}-C_{0}$, and local governments’ income is $R_{1}$.

Implementing the central government’s strategy or not depends on the central government’s punishment on local governments’ violation. If $R_{1}-P-S>R_{2}$, local governments still have incentives to violate the rules, and the central government’s policy is not well implemented. If $R_{1}-P-S<R_{2}$, the game can reach a Nash equilibrium of mixed strategy (supervision by the central government and no violation by local governments). For example, the implementation of the UGB policy in the fourteen pilot cities in China has significantly increased the cost of illegal land transfer by local governments, forcing them to obey the central government’s institutional arrangement on the allocation of land elements and choose the strategy of “no violation”. If $R_{1}-P-S=R_{2}$, local governments have no motivation to seek land finance directly, but when facing developers’ rent-seeking, local officials may take risks to conduct illegal behaviors (e.g., corruption) for their personal economic interests. Overall, the larger the $R_{1}-P-S-R_{2}$, the greater the probability of local governments’ violation. Accordingly, this study obtains Inference 1, which is as follows:

**Inference** **1.** *Under the situation in which the central government monitors the implementation of the UGB policy, the cost of transferring land elements by local governments in violation of regulations increases significantly, and “no violation” is regarded as their optimal strategy in the game with the central government*.

### 5.2. Game Analysis between Local Governments and the Real Estate Developer

#### 5.2.1. Model Assumptions

**Assumption** **4.** *Local governments have two goals. One is to promote local economy; the other is to gain personal interests of local government officials. The growth of local economy is usually associated with personal interests of officials, but the personal interests mentioned in this study refer to the economic benefit obtained by local officials through rent-seeking from the real estate developer when they have little hope in further promotion. After the market-oriented reform of land, there are legal land transfer methods such as bidding, auction, and listing in China’s primary land market. But the developer will seek rent from local governments in order to achieve particular interests and obtain land-use rights by manipulation. Therefore, local governments’ strategy choice set is legal transfer, illegal transfer; the strategy choice set of the developer is rent-seeking, no rent-seeking*.

**Assumption** **5.** *The only goal of the real estate developer is to maximize financial profits. Because there are many developers in the market, they have an incentive to seek rent in order to gain an advantage over local governments in monopolizing land supply. In this article, the model analysis only chooses one developer in the market with multiple developers of equal ability as an example, and the strategic choice for a single developer is rent-seeking or no rent-seeking. When local governments legally transfer land-use rights, the cost of rent-seeking by a developer is*$A_{1}$*, and when local governments violate the rules, the cost of rent-seeking by a developer is*$A_{2}$.

**Assumption** **6.** *Local governments obtain revenue from the transfer of land-use rights through legal means of bidding, auction, and listing is*$B_{0}$*, and the revenue obtained by the developer from the transfer of land in the secondary market is*$B_{1}$*. Local governments gain*$B_{3}$*for illegal land transfer*.

#### 5.2.2. Interest Matrix and Evolutionary Game Model

x represents the probability that the developer will choose not to seek rent, 1−x represents the probability that the developer will seek rent, y represents the probability of local governments’ legal transfer, and 1−y represents the probability of local governments’ illegal transfer. And the game profit matrix of local governments and the real estate developer is shown in Table 5.

(1) Dynamic equation of the real estate developer evolutionary game

The expected revenue and average revenue when a developer adopts the strategy of “no rent-seeking” and rent-seeking are $U_{11}$, $U_{12}$ and $U_{1}$, respectively, which are calculated as follows:

(7) $$\begin{array}{l} U_{11}=y(B_{1}-B_{0})\\ U_{12}=y(B_{1}-B_{0}-A_{1})+(1-y)(B_{1}-B_{3}-A_{2})\\ U_{1}=xU_{11}+(1-x)U_{12} \end{array}$$

The evolutionary game replication dynamic equation of probability x that the developer chooses the “no rent-seeking” strategy is:

(8) $$F_{K}(x)=dx/dt=x(U_{11}-U_{1})=x(1-x)(yB_{1}+A_{2}+yA_{2}+B_{3}-yB_{3}-B_{1})$$

(2) Dynamic equation of local governments evolutionary game

The expected income and average income when local governments adopt the strategy of “legal transfer” and “illegal transfer” are $U_{21}$, $U_{22}$ and $U_{2}$, respectively, and the calculations are as follows:

(9) $$\begin{array}{l} U_{21}=xB_{0}+(1-x)(B_{0}+A_{1})=(1-x)A_{1}+B_{0}\\ U_{22}=(1-x)(B_{3}+A_{2})\\ U_{2}=yU_{21}+(1-y)U_{22}=(1-x)(yA_{1}-yB_{3}-yA_{2}+B_{3}+A_{2})+yB_{0} \end{array}$$

The evolutionary game replication dynamic equation of the probability y that local governments choose the “legal sale” strategy is:

(10) $$F_{D1}(y)=dy/dt=y(U_{21}-U_{2})=y(1-y)((1-x)(A_{1}-B_{3}-A_{2})+B_{0}$$

Copy the dynamic equation system composed of Equations (8) and (10), as shown in Equation (11):

(11) $$\begin{array}{l} F_{K}(x)=dx/dt=x(U_{11}-U_{1})=x(1-x)(yB_{1}+A_{2}+yA_{2}+B_{3}-yB_{3}-B_{1})\\ F_{D1}(y)=dy/dt=y(U_{21}-U_{2})=y(1-y)((1-x)(A_{1}-B_{3}-A_{2})+B_{0}) \end{array}$$

(3) Evolutionary equilibrium analysis of cooperative subject behavior game

Let the equation value of the replicated dynamic Equation (11) be zero, and five equilibrium points are obtained as A (0, 1), B (0, 0), C (1, 0), D (1, 1), and E ($\left(A_{1}-B_{3}-A_{2}+B_{0})/(A_{1}-B_{3}-A_{2}),(B_{1}-A_{2}-B_{3})/(B_{1}+A_{2}-B_{3}\right)$).

The Jacobian matrix obtained from the replicated dynamic equations of Formula (11) is:

(12) $$\left[\begin{array}{cc} F1 & F2\\ F3 & F4 \end{array}\right]$$

The determinant of the matrix is det(J), and the trace of the matrix is tr(J). The stability analysis of the five equilibrium points is shown in Table 6 and Table 7.

The equilibrium points E, A, B, and C do not satisfy the conditions of det(J)>0 and tr(J)<0, so E ($\left(A_{1}-B_{3}-A_{2}+B_{0})/(A_{1}-B_{3}-A_{2}),(B_{1}-A_{2}-B_{3})/(B_{1}+A_{2}-B_{3}\right)$), A (0, 1), B (0, 0), and D (1, 1) do not meet the evolutionary stabilization strategy conditions. Only point D is the ESS point, and the system eventually converges to point D (1, 1), that is, the developer chooses not to seek rent and local governments sell land legally.

#### 5.2.3. Equilibrium Point Stability Analysis

Point D (1, 1) means the developer chooses not to seek rent, and the situation of local governments’ legal transfer is the expected state of the UGB policy. This situation usually occurs during the period of land policy implementation and regulation. Under the premise that the central government’s supervision is enhanced, the cost of local governments’ illegal transfers increased significantly. Under the premise of relevant policy constraints, local governments and the real estate developer regard legal behavior as an optimal strategy.

#### 5.2.4. Evolution Simulation Analysis

**Figure 7:**
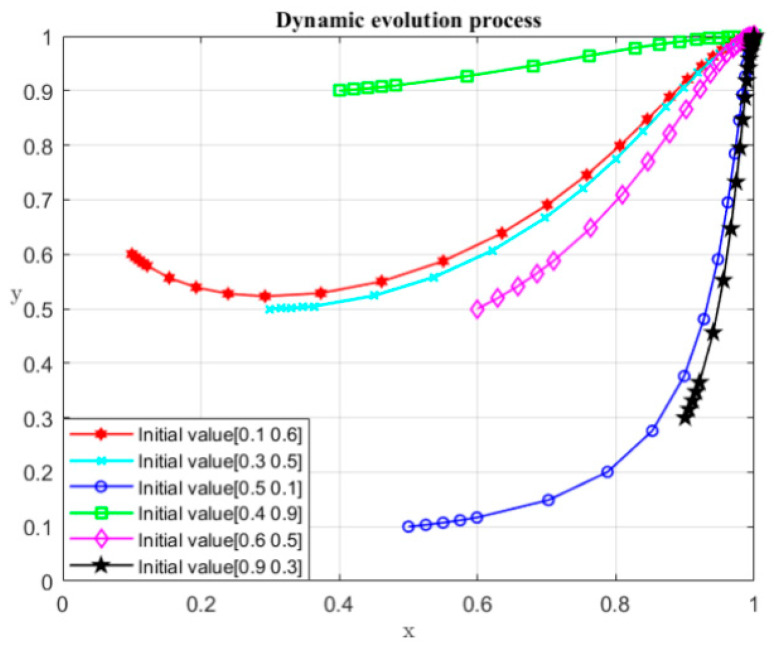
The dynamic evolution process (local governments and the real estate developer).

The definitions and assigned values of the relevant parameters are in Table 8, and the initial values (x,y) of the numerical simulation are set as (0.1, 0.6), (0.2, 0.8), (0.3, 0.5), (0.5, 0.5), (0.6, 0.5), (0.7, 0.2), (0.9, 0.3), and (0.7, 0.8). Figure 7 shows the dynamic evolution process of the participant strategy selection over time. It can be seen from Figure 7 that under the specified conditions, no matter what the initial values of x and y are, the outcome moves to point D (1, 1), which means the developer chooses not to seek rent, and the local governments legally sell land. Point D (1, 1) is the only equilibrium point in this case.

(1) When local governments operate illegally, the impact of the developer’s rent-seeking cost $A_{2}$ on the evolution results

**Figure 8:**
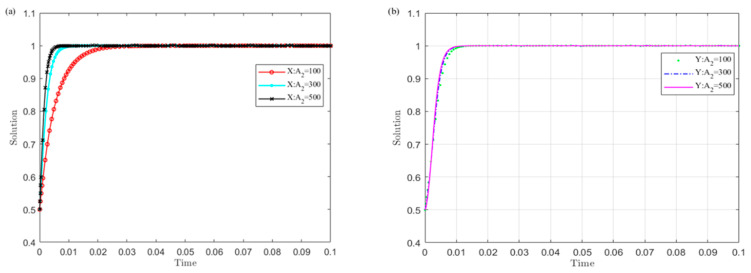
System strategy evolution results when $A_{2}=100,\;A_{2}=300,\;A_{2}=500$. (**a**) the real estate developer; (**b**) local governments.

Set $A_{2}$ as 100, 300, and 500, and simulate the evolution of the strategies of local governments and the developer. The results are shown in Figure 8.

The curves in Figure 8 show that when the local governments operate illegally, the larger the developer’s rent-seeking cost $A_{2}$, the sooner the developer converges to the no-rent-seeking strategy and local governments choose the legal selling strategy. Among them, when local governments operate illegally, the developer is more sensitive to changes in $A_{2}$ than local governments. This shows that increasing the developer’s rent-seeking cost is conducive to the successful implementation of the UGB policy.

(2) The influence of the income $B_{0}$ obtained by local governments from the legal transfer of land-use rights on the evolution results

**Figure 9:**
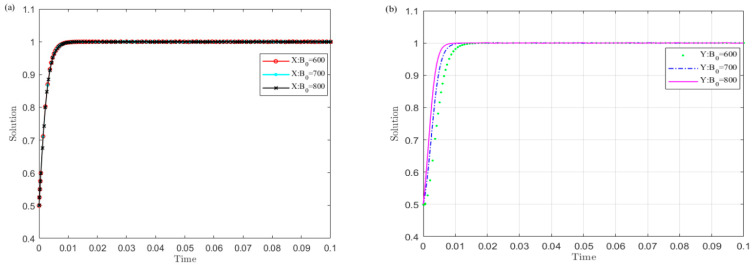
System strategy evolution results when $B_{0}=600,\;B_{0}=700,\;B_{0}=800$. (**a**) the real estate developer; (**b**) local governments.

Set $B_{0}$ as 600, 700, and 800, and simulate the evolution of the strategies of local governments and the developer. The results are shown in Figure 9.

The curves show that the larger the value $B_{0}$ of the income obtained by the local governments from the legal transfer of land-use rights, the sooner the developer converges to the no rent-seeking strategy and the governments choose the legal transfer strategy. Among them, local governments are more sensitive to the changes in $B_{0}$ than the developer. This shows that increasing the income obtained by the local governments from the legal transfer of land-use rights is conducive to the successful implementation of the UGB policy.

#### 5.2.5. Conclusions

Based on the payment matrix of local governments and the real estate developer (Table 5), it can be found that in the (legal transfer, no rent-seeking) strategy, local governments fully abide by the principles of land market transfer, legally and in compliance with the land transfer to the developer, who eventually obtain land-use rights through fair market competition. At this time, the revenue of local governments is $B_{0}$, and the land revenue obtained by the developer is $B_{1}-B_{0}$. In the (legal transfer, rent-seeking) strategy, due to information asymmetry, competition between developers is fierce. In order to obtain an edge over peers in local governments’ legal transfer of land, the developer still has the motivation of rent-seeking. Local governments’ revenue is $B_{0}+A_{1}$, and the developer’s revenue is $B_{1}-B_{0}-A_{1}$. In the (illegal transfer, no rent-seeking) strategy, local governments’ return is 0, and the developer’s return is 0. Finally, in the (illegal transfer, rent-seeking) strategy, local governments gain $B_{3}+A_{2}$, and the developer gains $B_{1}-B_{3}-A_{2}$.

In general, the higher $A_{1}$ is, the higher the probability of illegal land transfer by local governments is. The lower $B_{3}$ is, the greater the probability of rent-seeking is. In fact, when local governments’ strategy is legal sale, the developer’s strategy of rent-seeking has a yield of $B_{1}-B_{0}-A_{1}$, which is smaller than the yield of $B_{1}-B_{0}$ in the “no rent-seeking” strategy. Therefore, the developer’s optimal strategy is “no rent-seeking” in this case. Through similar analysis, it can be found that there are two Nash equilibria between local governments and the developer (legally transfer, no rent-seeking) and (illegally transfer, rent-seeking). The analysis in Section 4.1 showed that under the circumstance of central government supervision, the cost of illegal transfer by local governments will increase significantly, and the strategy of “no violation” has been regarded as the optimal strategy in the game with the central government. Accordingly, Inference 2 is obtained as follows:

**Inference** **2.** *In the case of the implementation of central supervision, legal transfer, no rent-seeking will become a Nash equilibrium solution to the game between local governments and the real estate developer*.

### 5.3. Game Analysis between Local Governments and Land-Expropriated Farmers

#### 5.3.1. Model Building

There are many conflicts between local governments and farmers whose land is expropriated. On the one hand, local governments expropriate rural land for the sake of public interests such as urban construction and economic development. Local governments need to consider not only farmers but also multiple stakeholders comprehensively. Therefore, the land expropriation arrangement of local governments may not fully meet the expectations of farmers. On the other hand, local governments have power in many departments and they can ignore social interests and act in line with department interests. However, given the fact that farmers will also maximize their interests, their living standard does not decline after land expropriation, and their awareness of rights protection keeps improving.

In the current land expropriation system, the compensation standard for farmers whose land is expropriated is relatively low. Local governments have no clear definition of public interests, the expropriation process is not standardized. The egoistic motivation of departments, the violent expropriation of land caused by power-for-benefit transaction, the forced demolition, and other problems are becoming increasingly serious. The existing land expropriation is an opaque decision-making process and gradually becomes a puppet of local officials, which seriously damages farmers’ right to know and right to participate in land expropriation. The farmers are a disadvantageous group of people, and they have difficulties in negotiating with local governments in land expropriation. They might resort to violent resistance. Therefore, the strategic choice set of local governments is reasonable compensation, and unreasonable compensation; the strategic choice set of land-expropriated farmers is cooperation and boycott.

#### 5.3.2. Model Assumptions

**Assumption** **7.** *When local governments offer reasonable compensation, local governments gain*$D_{1}$*from land expropriation, and the compensation for farmers whose land is expropriated is*$D_{2}$*. According to the current land management law and land expropriation system, even if reasonable compensation is offered, the compensation is equivalent to six to ten times the average annual output value of the cultivated land in the past 3 years. However, in the primary land trading market, local governments can sell the land-use right at a higher price and charge high land transfer fees, so*$D_{1}>D_{2}$.

**Assumption** **8.** *The added value of local governments’ unreasonable compensation is*$D_{3}$*, local governments are punished by the central government for unreasonable compensation in land expropriation*$E_{1}$*, and the cost of additional punishment if further social problems are caused is*$E_{2}$*. The cost of the land-expropriated farmers resisting local governments violation is*F*, and the benefit of successful resistance is*G.

#### 5.3.3. Interest Matrix and Evolutionary Game Model

x represents the probability that the land-expropriated farmers will choose to cooperate, 1−x represents the probability that the farmers will not cooperate, y represents the probability of reasonable compensation by local governments, and 1−y represents the probability of unreasonable compensation. And the game profit matrix of local governments and land-expropriated farmers is shown in Table 9.

(1) Evolutionary game dynamic equation of the land-expropriated farmers

The expected income and average income when the land-expropriated farmers N adopt the strategy of “cooperation” and “boycott” are $U_{11}$, $U_{12}$ and $U_{1}$ respectively, and the calculation is as follows:

(13) $$\begin{array}{l} U_{11}=yD_{2}+(1-y)D_{2}=D_{2}\\ U_{12}=y(D_{2}-F)+(1-y)(D_{2}-F+G)=D_{2}-F+(1+y)G\\ U_{1}=xU_{11}+(1-x)U_{12}=D_{2}+(1-x)(G+yG-F) \end{array}$$

The evolutionary game replication dynamic equation of the probability x that the land-expropriated farmers choose the “cooperation” strategy is:

(14) $$F_{N}(x)=dx/dt=x(U_{11}-U_{1})=x(1-x)(F-G+yG)$$

(2) Dynamic equation of evolutionary game of local governments

Local governments can adopt the “reasonable compensation” strategy or the “unreasonable compensation” strategy, the expected income and average income are $U_{21}$, $U_{22}$, and $U_{2}$ respectively, and the calculation is as follows:

(15) $$\begin{array}{l} U_{21}=xD_{1}+(1-x)D_{1}=D_{1}\\ U_{22}=x(D_{1}+D_{3}-E_{1})+(1-x)(D_{1}+D_{3}-E_{1}-E_{2})=D_{1}+D_{3}-E_{1}+(x-1)E_{2}\\ U_{2}=yU_{21}+(1-y)U_{22}=(1-y)(D_{3}-E_{1}+xE_{2}-E_{2})+D_{1} \end{array}$$

The evolutionary game replication dynamic equation of the probability *y* that local governments choose the “reasonable compensation” strategy is:

(16) $$F_{D2}(y)=dy/dt=y(U_{21}-U_{2})=y(1-y)(E_{1}+E_{2}-xE_{2}-D_{3})$$

Copy the dynamic equation system composed of Equations (14) and (16), as shown in Equation (17):

(17) $$\begin{array}{l} F_{N}(x)=dx/dt=x(U_{11}-U_{1})=x(1-x)(F-G+yG)\\ F_{D2}(y)=dy/dt=y(U_{21}-U_{2})=y(1-y)(E_{1}+E_{2}-xE_{2}-D_{3}) \end{array}$$

(3) Evolutionary equilibrium analysis of cooperative subject behavior game

Let the equation value of the replicated dynamic Equation (17) be zero, and five equilibrium points are obtained as A (0, 1), B (0, 0), C (1, 0), D (1, 1), and E $\left((E_{1}+E_{2}-D_{3})/E_{2},(G-F)/G\right)$.

The Jacobian matrix obtained from the replicated dynamic equations of Formula (17) is as follows:

(18) $$\left[\begin{array}{cc} F1 & F2\\ F3 & F4 \end{array}\right]$$

The determinant of the matrix is det(J), the trace of the matrix is tr(J), and the stability analysis of the five equilibrium points is shown in Table 10 and Table 11.

The value of the trace of the equilibrium point E is 0, and the equilibrium point A does not satisfy the conditions of det( J)>0 and tr(J)<0.

Therefore, E ($C_{1}/(P+S)-1,(R_{2}-R_{1})/(S+P)+1$) and A (0, 1) do not satisfy the evolutionary stable strategy condition. The remaining three equilibrium points satisfy the condition as local stable points are shown in Table 11.

#### 5.3.4. Equilibrium Point Stability Analysis

(1) B (0, 0)

Point B (0, 0) means local governments offer unreasonable compensation and the land-expropriated farmers do not cooperate. When F<G and $E_{1}+E_{2}<D_{3}$, B (0, 0) is the only equilibrium point. This situation usually occurs in the early stage of the implementation of the land policy, when local governments do not clearly define public interests, and problems such as irregular land acquisition procedures, department egoistic motivation, and power-for-benefit transactions arise. These can also be accompanied by violent land acquisition and intensified forced demolition. The land-expropriated farmers are disadvantaged, and it is difficult for them to negotiate with the government on land expropriation. They may take violent resistance and non-cooperative actions.

Point B (0, 0) means the unreasonable compensation by local governments and the boycott of the land-expropriated farmers. Point B (0, 0) is not the expected outcome of the UGB policy implementation. To reverse this situation, $E_{1}$ and $E_{2}$ must be increased and $D_{3}$ should be decreased. In practice, the central government should not only increase the penalties on local governments’ illegal operations, but also increase the cost of additional penalties on local governments for potential social problems stemming from land expropriation.

(2) C (1, 0)

Point C (1, 0) means local governments offer reasonable compensation, and the land-expropriated farmers do not cooperate. When G<F and $E_{1}<D_{3}$, Point C (1, 0) is the only equilibrium point. This situation generally occurs during the period of concern on the implementation of the land policy. On the one hand, the living cost for the land-expropriated farmers is still high, and the farmers will keep fighting for more benefit; the cost F of the land-expropriated farmers resisting land expropriation is relatively low, and the income G is relatively high. In this case, the famer’s income of boycott is higher than the reasonable compensation offered by local governments.

(3) D (1, 1)

Point D (1, 1) means the local governments offer reasonable compensation and the land-expropriated farmers cooperate. When $D_{3}<E_{1}$, it is the only equilibrium point. This situation generally occurs during the implementation period of the land policy, and local governments’ compensation for farmers whose land has been expropriated is improved, and the land expropriation system is feasible and effective. In this case, the relevant benefit obtained by the land-expropriated farmers from cooperation with local governments is higher than that obtained by boycotting. At this point, the land-expropriated farmers cooperate with local governments. This point is a win-win solution.

#### 5.3.5. Evolutionary Simulation Analysis

**Figure 10:**
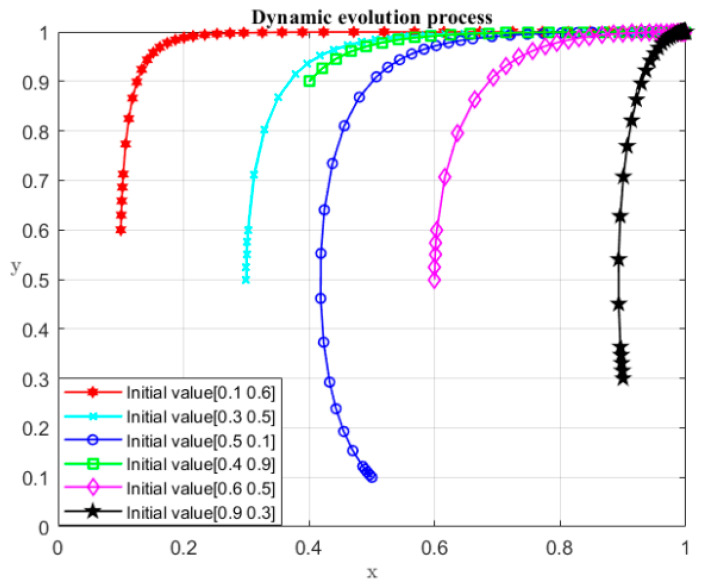
System evolution results when $D_{3}<E_{1}$ (local governments and land-expropriated farmers).

It can be seen from the above game process that when $D_{3}<E_{1}$, the system evolves to the desired equilibrium point (1, 1). The definitions and assigned values of the relevant parameters are shown Table 12, and the initial values (x, y) of the numerical simulation are respectively set as (0.1, 0.6), (0.2, 0.8), (0.3, 0.5), (0.5, 0.5), (0.6, 0.5), (0.7, 0.2), (0.9, 0.3), and (0.7, 0.8). Figure 10 shows the dynamic evolution process of participant strategy selection over time. It can be seen from Figure 10 that under the specified conditions, no matter what the initial values of x and y are, the system converges to (1, 0), which means the local governments do not violate the regulations and the central government also monitors local governments.

(1) The influence of the unreasonable compensation of local governments on the evolution result of the added value $D_{3}$

**Figure 11:**
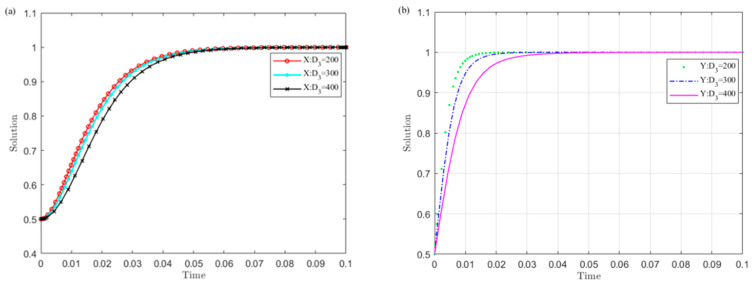
Evolution results of system strategy when $D_{3}=200,\;D_{3}=300,\;D_{3}=400$. (**a**) land-expropriated farmers; (**b**) local governments.

Set $D_{3}$ as 200, 300, 400, and simulate the strategy evolution of the land-expropriated farmers and local governments. The results are shown in Figure 11.

From curves in Figure 11, we can see that the smaller the added value $D_{3}$ of unreasonable compensation by local governments, the faster local governments converge to the reasonable compensation strategy. Among them, local governments are more sensitive to the changes in $D_{3}$ than the land-expropriated farmers. This shows that reducing the added value of unreasonable compensation by local governments is conducive to the successful implementation of the UGB policy.

(2) The impact of central government penalties $E_{1}$ for local governments’ unreasonable compensation on evolutionary results

**Figure 12:**
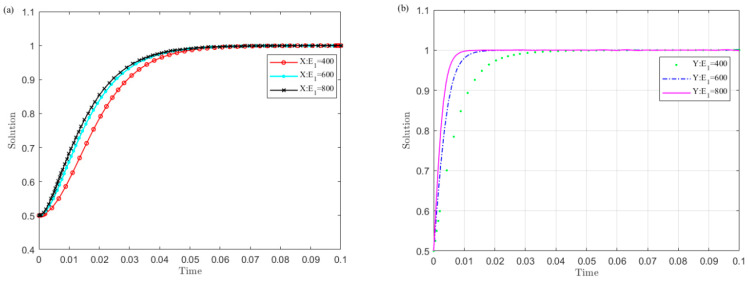
System strategy evolution results when $E_{1}=400,\;E_{1}=600,\;E_{1}=800$. (**a**) land-expropriated farmers; (**b**) local governments.

Set $E_{1}$ as 400, 600, 800, and simulate the strategy evolution of the land-expropriated farmers and local governments. The results are shown in Figure 12.

Figure 12 shows that local governments will be punished by the central government for their unreasonable compensation. The larger the value of $E_{1}$ is, the sooner local governments converge to a reasonable compensation strategy. Among them, if the local governments offer unreasonable compensation, it will be punished by the central government. This shows that reducing the added value of unreasonable compensation by local governments is conducive to the successful implementation of the UGB policy.

#### 5.3.6. Conclusions

Based on the payment matrix of local governments and the land-expropriated farmers (Table 9), several conclusions can be drawn. In the strategy of reasonable compensation and cooperation, local governments make the land-expropriated farmers actively cooperate through reasonable compensation. At this point, local governments’ income is $D_{1}$, and the land-expropriated farmers’ income is $D_{2}$. In the (reasonable compensation, boycott) strategy, because local governments try to maximize the overall social welfare while the land-lost farmers maximize their own interests on land expropriation, reasonable compensation may not meet the expectation of land-lost peasants. The resulting boycott forces local governments to offer reasonable compensation and thus have revenue $D_{1}$, while the income of the land-lost farmers is $D_{2}-F$. In this sense, the (unreasonable compensation, cooperation) strategy is the most desired result for local governments, and local governments can obtain $D_{1}+D_{3}-E_{1}$, and the farmers whose land is expropriated can obtain $D_{2}$.

In the strategy of unreasonable compensation and boycott, the unreasonable compensation of local governments is strongly resisted by the farmers whose land is expropriated. The farmers can take measures to reject the unreasonable compensation and make the violation of local governments detected via media and other channels. As a result, local governments are more likely to be punished for their unreasonable compensation. Therefore, the income of local governments is $D_{1}+D_{3}-E_{1}-E_{2}$, and the income of the farmers is $D_{2}-F+G$.

Only when the famer’s cost of boycott is low and the benefit is high can the reasonable compensation be realized. In the UGB situation for local governments the cost of taking the “unreasonable compensation” strategy continues to grow with intensified regulation and greater punishment from the central government. In other words, the central government should raise the punishment $E_{1}$ over the added value of local governments’ violation $D_{3}$. Accordingly, Inference 3 is obtained as follows:

**Inference** **3.** *In the case of the implementation of the central supervision policy such as the UGB policy, even if the strategy of land-expropriated farmers is “cooperation” or “boycott”, local governments always take “reasonable compensation” as its optimal strategy*.

## 6. Discussion and Conclusions

Land finance constitutes a strong support for economic growth, but excessive dependence on land finance will eventually evolve into a negative constraint on economic growth. In fact, land finance increases the fiscal revenue of local governments, promotes technology, and strengthens the spillover effect of capital [61] and human resources through urban industrialization and industrial structure adjustment. In this sense, land finance is an important determinant of regional economic growth. Dependence on land finance reflects the structural characteristics of fiscal revenue of local governments [62]. When land finance contributes to fiscal revenue to a proper extent, it promotes economic growth. However, excessive dependence on land finance leads to an imbalanced financial revenue structure. Due to the limited supply of land, land finance is not a sustainable power for economic growth. Therefore, local governments should stick to a moderate level of land finance and adjust the structure of fiscal revenue rather than passively and increasingly rely on it. Hence, local governments should also keep seeking other sustainable sources of fiscal revenue.

Because of the imbalance between cost and benefit, Chinese local governments have significant incentives on land fiscal. In a sense, the pursuit of land finance has led to many problems such as the blind expansion of urban space in China, the low efficiency of urban land use, the large city scales and the low efficiency of urban operation. Restricting land finance can improve the quality of urbanization and lead a real estate market that is more rational. After the implementation of the UGB policy, urban land capacity will be determined, which will break the situation of “unlimited land capacity” faced by land finance in the past, and the cost of land finance acquisition will also rise. All of these restrict the dependence on land finance among local governments and encourage them to seek new sources of fiscal revenue. Since the UGB policy will impose strong constraints on land finance, it will also regulate urban layout and industrial layout. Considering local governments that are constrained by the UGB policy also benefit from the land fiscal, this duality determines that stakeholders will show a typical game process during the implementation of the UGB policy.

The effective implementation of the UGB policy, by controlling urban land capacity, can logically be a solution to the land finance dependence dilemma of local governments. In fact, the essence of the UGB policy is a set of technical means and policy tools to control urban sprawl. By limiting urban land capacity in a given period, it will mitigate the “unlimited land capacity” situation faced by local governments in the past and become a new idea to solve the dilemma of land finance dependence. The formation of the dilemma is not only affected by the tax-sharing reform, urban-rural dual land system and other relevant institutional arrangements, but also the political performance competition among local governments [60]. On the one hand, through large-scale urban land transfer and space development, local governments rely on land to attract more investment in the competition with other cities, which leads to a land exploitation competition. On the other hand, the income generated from urban land transfer or land lease has become an important financing channel for local governments to carry out urban infrastructure construction. The benefit easily obtained from land finance makes local governments compose ambitious investment plans and raise the debt level and default risk of local governments. The UGB policy can restrict urban land supply and raise local governments’ cost in land finance. Stronger constraints on land finance push local governments to optimize their fiscal revenue structure, contributing to sustainable fiscal revenue sources and a better land use mechanism.

The UGB policy contributes to the coordination mechanism for the interests among land finance stakeholders and provides an important institutional foundation for improving welfare for all. In the process of land finance operation, the central government, local governments, real estate developers, and farmers have different choices and strategies, and thus constitute a complex and interactive game relationship. Once the economic development excesses a certain level, the central government’s emphasis on efficiency will lead to an imbalanced regional economic development. Then, the focus of the central government will be moved from economic growth to the optimization of social resource allocation. The UGB policy becomes an important cornerstone for constructing the stakeholder coordination mechanism and maximizing social welfare. The UGB policy is also an important channel through which the central government can manage and supervise local governments. It is bound to increase the relevant cost of local governments’ land acquisition, reduce the dependence of local governments on land finance, and increase the cost of rent-seeking by real estate developers. Therefore, the implementation of the UGB policy can lead to significant changes in the actual benefit distribution among stakeholders in land finance. It also pushes local governments and other stakeholders to adjust their strategies, and provides a possible solution to the dilemma of LFD.

It is noteworthy that as an institutional arrangement to solve the dilemma of land fiscal dependence, the successful implementation of the UGB policy requires the improvement in relevant supporting measures, such as optimizing the performance assessment mechanism of local government officials, reforming the institutional arrangement of the one-off land transfer fee, and realizing a proper allocation of duties among governments of different administrative levels. Moreover, deepening the reform of the rural land expropriation compensation system is also important. Local governments should never ignore the “autonomy” of the farmers cohort in this process.

In fact, the approach of evolutionary game simulation has been widely utilized in the discussion process of urban land use. Game theory can effectively characterize the process of real-world brownfield redevelopment negotiations [63] and it becomes an effective theory to explain the land redevelopment process [64]. Moreover, because the evolutionary game simulation approach considers realistic conflicts in the collective decision-making process, it is considered to be effective for the analysis in urban land spatial planning [65]. The assumptions in this study are based on the realities of China, i.e., the behaviors that various stakeholders may take in reality under a highly constrained or deregulated power system. However, due to the stringent assumptions made in this study, the applicability of this research may have certain limitations; however, it still provides an important analytical framework for a deep understanding of the corrective effect of the UGB policy on LFD of local governments in China. Future studies can consider introducing econometric models with the municipal panel data to estimate the actual impact of the UGB policy on Chinese cities.

## Figures and Tables

**Table 1:** The game profit matrix (the central government and local governments).

	Local Governments Do Not Violate	Local Governments Violate
Central Government does not Monitor	$(R_{0},R_{2}$)	$(R_{0}-C_{0},R_{1}$)
Central Government Monitors	$(R_{0}-C_{1},R_{2}$)	$(R_{0}-C_{0}-C_{1}+P+S,R_{1}-P-S$)

**Table 2:** Numerical expression of stable points (the central government and local governments).

Equilibrium	det(J)	tr(J)
A (0, 1)	$\left(R_{2}-R_{1})\times (P+S-C_{1}\right)$	$R_{2}-R_{1}+P+S-C_{1}$
B (0, 0)	$\left(R_{2}-R_{1})\times (C_{1}-P+S\right)$	$R_{2}-R_{1}-P-S+C_{1}$
C (1, 0)	$\left(R_{1}-R_{2}-S-P)\times (C_{1}-2P-2S\right)$	$R_{1}-R_{2}+C_{1}-3P-3S$
D (1, 1)	$\left(R_{1}-R_{2})\times (2P+2S-C_{1}\right)$	$R_{1}-R_{2}+2P+2S-C_{1}$
$E\;(\begin{array}{l} C_{1}/(P+S)-1,\\ (R_{2}-R_{1})/(S+P)+1 \end{array})$	$\begin{array}{l} (C_{1}-P-S)\times (C_{1}-2P-2S)\times \\ \left(R_{2}-R_{1})\times (R_{2}-R_{1}+P+S)/(P+S\right) \end{array}$	0

**Table 3:** Conditions for each equilibrium point to become a local stable point (the central government and local governments).

Equilibrium	Condition
A (0, 1)	$P+S<C_{1}$
C (1, 0)	$R_{1}-P-S<R_{2},C_{1}<2P+2S$

**Table 4:** Parameter meaning and assignment (the central government and local governments).

Parameter	Meaning	Numerical Value
$R_{0}$	Central government revenue	1000
$R_{1}$	Local governments’ illegal operation income	600
$R_{2}$	Benefit from the implementation of the relevant policy by local governments	400
$C_{0}$	The cost paid by the central government to address the negative externalities arising from local governments’ violation	200
$C_{1}$	The cost of monitoring by the central government to local governments	200
P	Central government’s punishment of local governments for illegal operations	300
S	Central government confiscates local governments’ illegal earnings	400

**Table 5:** Game profit matrix (local governments and the real estate developer).

	The Developer Does Not Seek Rent	The Developer Seeks Rent
Legal Transfer by Local Governments	$\left(B_{0},B_{1}-B_{0}\right)$	$\left(B_{0}+A_{1},B_{1}-B_{0}-A_{1}\right)$
Illegal Transfer by Local Governments	(0,0)	$\left(B_{3}+A_{2},B_{1}-B_{3}-A_{2}\right)$

**Table 6:** Numerical expression of stable points (local governments and the real estate developer).

Equilibrium	det(J)	tr(J)
A (0, 1)	$2A_{2}\times \left(A_{2}+B_{3}-A_{1}-B_{0}\right)$	$3A_{2}+B_{3}-A_{1}-B_{0}$
B (0, 0)	$\left(A_{2}+B_{3}-B_{1}\right)\times \left(A_{1}-B_{3}-A_{2}+B_{0}\right)$	$A_{1}+B_{0}-B_{1}$
C (1, 0)	$\left(B_{1}-A_{2}-B_{3}\right)\times B_{0}$	$B_{0}+B_{1}-A_{2}-B_{3}$
D (1, 1)	$2A_{2}B_{0}$	$-2A_{2}-B_{0}$
$E(\begin{array}{l} \left(A_{1}-B_{3}-A_{2}+B_{0})/(A_{1}-B_{3}-A_{2}\right)\\ ,(B_{1}-A_{2}-B_{3})/(B_{1}+A_{2}-B_{3}) \end{array})$	$\begin{array}{l} -A_{2}B_{0}\times \left(A_{1}-B_{3}-A_{2}+B_{0}\right)\times \\ \left(B_{1}-A_{2}-B_{3}\right)/\left(A_{1}-B_{3}-A_{2}\right) \end{array}$	0

**Table 7:** Evolutionary stability points (local governments and the real estate developer).

Equilibrium Points	det(J) Notation	tr(J) Notation	Equalization Results
A (0, 1)	+	+	Unstable Point
B (0, 0)	–	+	Saddle Point
C (1, 0)	–	X	Saddle Point
D (1, 1)	+	–	ESS
$E\;(\begin{array}{l} \left(A_{1}-B_{3}-A_{2}+B_{0})/(A_{1}-B_{3}-A_{2}\right)\\ ,(B_{1}-A_{2}-B_{3})/(B_{1}+A_{2}-B_{3}) \end{array})$	+	0	Saddle Point

**Table 8:** Parameter meaning and assignment (local governments and the real estate developer).

Parameter	Meaning	Numerical Value
$A_{1}$	When the local governments legally assign the land-use rights, the developer’s rent-seeking cost	100
$A_{2}$	When local governments operate illegally, the developer’s rent-seeking cost	200
$B_{0}$	The income obtained by the local governments through the legal means of recruiting, auctioning, and listing the land-use rights	700
$B_{1}$	The land income obtained by the developer through the transfer of the secondary land market, etc.	500
$B_{3}$	Proceeds from the illegal sale of land by local governments	900

**Table 9:** Game profit matrix (local governments and land-expropriated farmers).

	The Land-Expropriated Farmers Cooperate	The Land-Expropriated Farmers Does Not Cooperate
Reasonable Compensation by Local Governments	$(D_{1},D_{2}$)	$(D_{1},D_{2}-F$)
Unreasonable Compensation by Local Government	$(D_{1}+D_{3}-E_{1},D_{2}$)	$(D_{1}+D_{3}-E_{1}-E_{2},D_{2}-F+G$)

**Table 10:** Numerical expression of stable points (local governments and land-expropriated farmers).

Equilibrium	det(J)	tr(J)
A (0, 1)	$F\times (D_{3}-E_{1}-E_{2})$	$F+D_{3}-E_{1}-E_{2}$
B (0, 0)	$\left(F-G)\times (E_{1}+E_{2}-D_{3}\right)$	$F-G+E_{1}+E_{2}-D_{3}$
C (1, 0)	$\left(G-F)\times (E_{1}-D_{3}\right)$	$G-F+E_{1}-D_{3}$
D (1, 1)	$-F\times (D_{3}-E_{1})$	$-F+D_{3}-E_{1}$
$E\;((E_{1}+E_{2}-D_{3})/E_{2},(G-F)/G$)	$F\times (G-F)(E_{1}+E_{2}-D_{3})(D_{3}-E_{1})/E_{2}G$	0

**Table 11:** Conditions for each equilibrium point to become a local stable point (local governments and land-expropriated farmers).

Equilibrium	Condition
B (0, 0)	$F<G,E_{1}+E_{2}<D_{3}$
C (1, 0)	$G<F,E_{1}<D_{3}$
D (1, 1)	$D_{3}<E_{1}$

**Table 12:** Parameter meaning and assignment (local governments and land-expropriated farmers).

Parameter	Meaning	Numerical Value
$D_{1}$	Local governments’ income after reasonable compensation	1000
$D_{2}$	The reasonable compensation received by the farmers	400
$D_{3}$	Income added value of unreasonable compensation by local governments	300
$E_{1}$	Central government’s penalties on local governments for their unreasonable compensation from the central government	500
$E_{2}$	Additional penalties on local governments for future potential social problems caused by their unreasonable compensation	200
F	The cost of the land-expropriated farmers resisting local governments’ violation	100
G	Gains of the land-expropriated farmers for successful boycott against local governments’ violation	200

## Data Availability

Not applicable.
